# Supplementary material for: The relationship between advanced glycation end products and gestational diabetes: A systematic review and meta-analysis
Source: PLoS One. 2020 Oct 21;15(10):e0240382. doi: 10.1371/journal.pone.0240382 (PMC7577486; doi:10.1371/journal.pone.0240382)
Supplement: S3 Table — The table shows the risk of bias assessments of studies with regard to design, conduct and analysis. (DOCX) [file pone.0240382.s003.docx]

**S3 Table. Quality assessment of studies using JBI's critical appraisal tools designed for case-control and cross-sectional studies**

|  | Studies | JBI's critical appraisal questions | | | | | | | | | | Overall score |
| --- | --- | --- | --- | --- | --- | --- | --- | --- | --- | --- | --- | --- |
|  |  | Q1 | Q2 | Q3 | Q4 | Q5 | Q6 | Q7 | Q8 | Q9 | Q10 |  |
| Case-control studies (out of 10) | | | | | | | | | | | | |
|  | Aziz et al | Y | Y | Y | Y | Y | Y | Y | Y | U | Y | 9 |
|  | Bartakova et al | Y | Y | Y | Y | Y | U | U | Y | U | Y | 7 |
|  | Cosson et al | Y | Y | Y | Y | Y | U | U | Y | N | Y | 7 |
|  | Davison et al | U | U | Y | Y | Y | N | Y | Y | Y | Y | 7 |
|  | Guosheng et al | Y | Y | Y | Y | Y | U | U | Y | N | Y | 7 |
|  | Harsem et al | Y | N | Y | Y | Y | Y | N | Y | U | Y | 7 |
|  | Li et al | Y | Y | Y | Y | Y | Y | Y | Y | U | Y | 9 |
|  | Li and Yang | Y | Y | Y | Y | Y | Y | Y | Y | U | Y | 9 |
|  | Krishnasamy et al | Y | Y | Y | Y | Y | Y | Y | Y | U | Y | 9 |
|  | Krishnasamy et al' | Y | U | U | Y | Y | N | N | Y | U | Y | 5 |
|  | Lobo et al | Y | Y | Y | Y | Y | Y | Y | N | U | Y | 8 |
|  | Mai et al | Y | Y | Y | Y | Y | Y | Y | Y | Y | Y | 10 |
|  | Pertyn´ ska-Marczewska | Y | Y | Y | N | Y | Y | Y | N | U | Y | 7 |
| Cross-sectional studies (out of 9) | | | | | | | | | | | | |
|  | Boutzios et al | Y | Y | N | Y | Y | Y | Y | Y | Y | --- | 8 |
|  | Morales et al | N | Y | N | N | N | Y | Y | Y | Y | --- | 5 |
|  | de Ranitiz-Greven et al | Y | Y | Y | Y | Y | Y | Y | Y | Y | --- | 9 |

Y, yes; N, No; U, unclear; Q, question. Overall score is calculated by counting the number of Ys in each row.

*NB: The respective order of questions and their details is available online for both studies*
