# Supplementary material for: The relationship between advanced glycation end products and gestational diabetes: A systematic review and meta-analysis
Source: PLoS One. 2020 Oct 21;15(10):e0240382. doi: 10.1371/journal.pone.0240382 (PMC7577486; doi:10.1371/journal.pone.0240382)
Supplement: Attachment — Submitted filename: Response to Reviewers.docx [file pone.0240382.s004.docx]

**Response to Reviewers**

**Dear Editor,**

Thank you for your constructive comments for updating this manuscript.

Here are the changes we made in the body of the manuscript during revision.

- The reviewer recommended updating the manuscript with latest (2020) references. Accordingly, we incorporated six relevant references in the background and discussion sections including the one recommended by the reviewer. We have accepted the reviewer insights in amending the manuscripts to date. Kindly check the track changed manuscript, please.
- We have also undergone careful language (mainly punctuation, syntax and normalization issues) and technical editions throughout the manuscript to make it clearer and easier to comprehend
- We have consistently formatted the ‘texts within figures’ to avoid ambiguity
- We have strictly followed PLOS ONE formatting guidelines including
  - Correcting all figures using PACE (e.g. tif compatible format) with better resolution
  - Rearranging the position of tables next to the paragraphs that they are first cited
  - Formatting the title page, heading and subheadings of main text as per the guideline
  - Formatting the references consistently as per the PLOS ONE style (e.g. Vancouver with six authors followed by et al….)
- The PRISMA flow diagram was followed as per the standard
- The full search strategy for legitimate databases such as PubMed, MEDLINE, and EMBASE was clearly and concisely presented in the methodology and reported in the PRISMA checklist.
- PRISMA Checklist was updated for the revised manuscript and all the contents of the checklist were adequately addressed in this systematic review and meta-analysis
- We have further elaborated the eligibility criteria
- The final formatting including rearrangement of tables and figures was made in the clean version only (labeled as manuscript)
- We have amended the title in the manuscript and online platform to be identical

**Regards,**

**Authors**
